# Oxygen-Terminated (1 × 1) Reconstruction of Reduced Magnetite Fe_3_O_4_(111)

**DOI:** 10.1021/acs.jpclett.3c00281

**Published:** 2023-03-28

**Authors:** Florian Kraushofer, Matthias Meier, Zdeněk Jakub, Johanna Hütner, Jan Balajka, Jan Hulva, Michael Schmid, Cesare Franchini, Ulrike Diebold, Gareth S. Parkinson

**Affiliations:** † Institute of Applied Physics, Technische Universität Wien, Wiedner Hauptstraße 8-10/E134, 1040 Wien, Austria; ‡ University of Vienna, Faculty of Physics and Center for Computational Materials Science, 1090 Wien, Austria; § Alma Mater Studiorum, Università di Bologna, 40127 Bologna, Italy

## Abstract

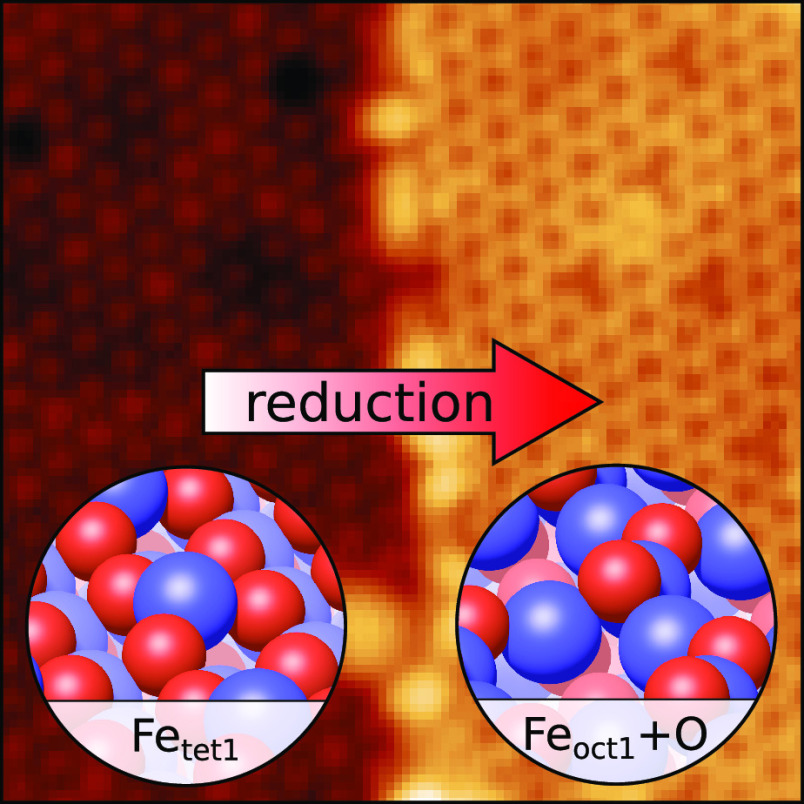

The (111) facet of magnetite (Fe_3_O_4_) has been studied extensively by experimental and theoretical methods, but controversy remains regarding the structure of its low-energy surface terminations. Using density functional theory (DFT) computations, we demonstrate three reconstructions that are more favorable than the accepted Fe_oct2_ termination under reducing conditions. All three structures change the coordination of iron in the kagome Fe_oct1_ layer to be tetrahedral. With atomically resolved microscopy techniques, we show that the termination that coexists with the Fe_tet1_ termination consists of tetrahedral iron capped by 3-fold coordinated oxygen atoms. This structure explains the inert nature of the reduced patches.

Magnetite (Fe_3_O_4_) is extremely common in nature and is an important catalyst material.^1−3^ While the surface structure of the (001) facet is well-understood,^3,4^ the lowest-energy Fe_3_O_4_(111) surface remains controversial despite decades of study. A key issue is that multiple terminations often coexist, depending on both the preparation conditions and the sample history. This complicates the interpretation of area-averaging methods^5,6^ and necessitates the use of local probes such as scanning tunneling microscopy (STM). Many atomically resolved STM images of UHV-prepared samples have been published, but questions remain, particularly about the structures formed under reducing conditions.

Samples annealed in oxygen-rich conditions (*p*_O_2__ ≈ 10^–6^ mbar, *T* = 870–1000 K^7−11^) usually exhibit a hexagonal array of protrusions with a nearest neighbor distance of 5.9 Å. Today, it is generally accepted that this corresponds to a relaxed bulk-truncation at the Fe_tet1_ plane (see Figure 1 for layer labeling and a top view of the Fe_tet1_ structure).^7−9,12^ This surface typically coexists with areas of a second (1 × 1)-periodic honeycomb structure. This has been attributed to an Fe_oct2_ termination,^11,13^ which DFT calculations suggest becomes more stable than the Fe_tet1_ termination under reducing conditions. A long-range ordered structure known as the “biphase” termination emerges under extremely reducing conditions, and this has been interpreted as either islands of Fe_1–*x*_O(111) coexisting with magnetite^14−17^ or as a moiré pattern formed by an FeO-like terminating layer.^18^

**Figure 1:**
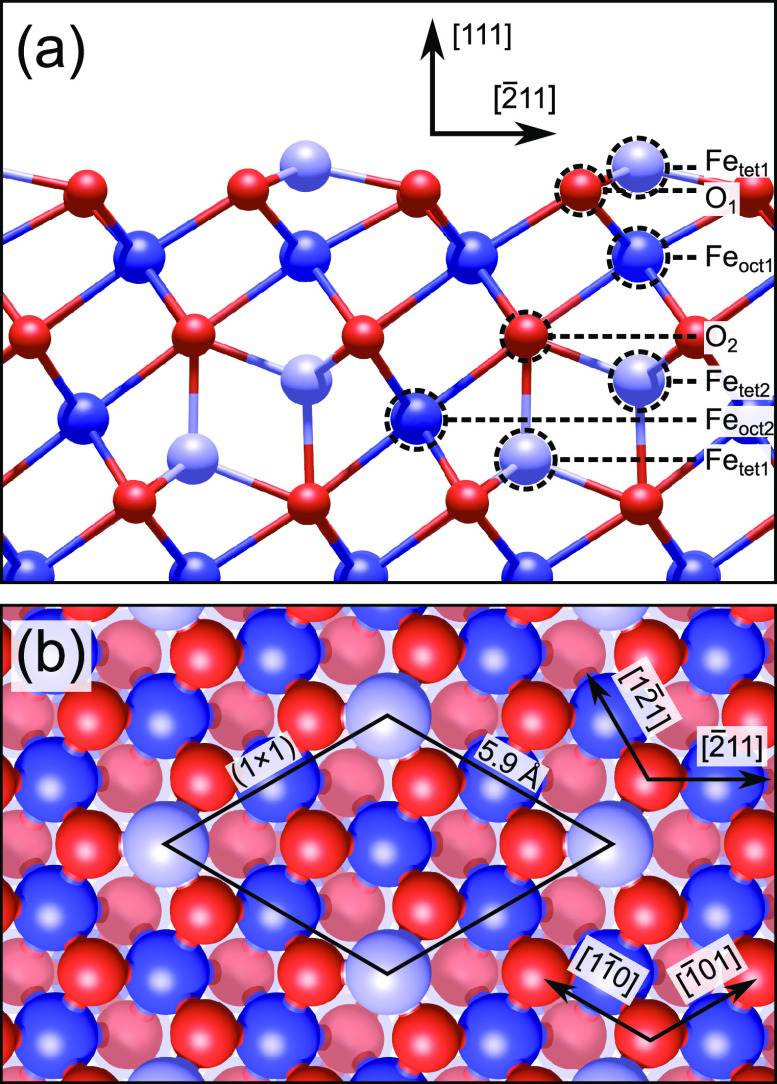
Fe_3_O_4_(111) Fe_tet1_ termination in (a) side and (b) top view. Tetrahedrally coordinated iron is light blue, octahedrally coordinated iron is dark blue, and oxygen is red. Oxygen in the deeper O_2_ layer is pale red in (b). The layer naming convention is indicated in (a), and a (1 × 1) unit cell is drawn in (b).

In this Letter, we introduce a revised phase diagram of Fe_3_O_4_(111) featuring three new terminations that are more stable than the Fe_oct2_ surface under reducing conditions. On the basis of noncontact atomic force microscopy (ncAFM) images, we assign the honeycomb patches observed experimentally to a termination at the Fe_oct1_ plane with an additional oxygen capping layer.

Figure 2 shows the updated surface phase diagram of Fe_3_O_4_(111) based on our DFT+*U* calculations. Black lines correspond to the most favorable terminations published previously,^10,19^ and colored lines correspond to the new models introduced here. We find three (1 × 1)-periodic reconstructions to be favorable over the existing models under reducing conditions. The corresponding atomic models are shown in Figure 3.

**Figure 2:**
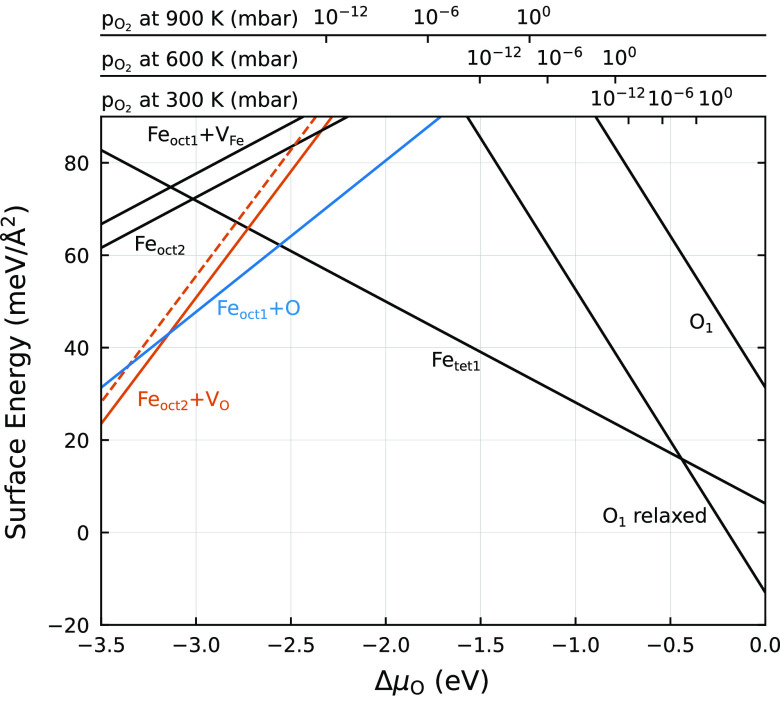
Surface energies of different terminations as a function of the oxygen chemical potential Δμ_O_. The top axes indicate the corresponding oxygen partial pressures at three temperatures. Colored lines are new models introduced here, and black lines correspond to terminations considered in previous work. “O_1_ relaxed” is the modified O_1_ termination introduced in ref (10), while all other models can be found in ref (19). The dashed orange line corresponds to the Fe_oct2_+V_O_ termination with a registry shift, as discussed in the text.

**Figure 3:**
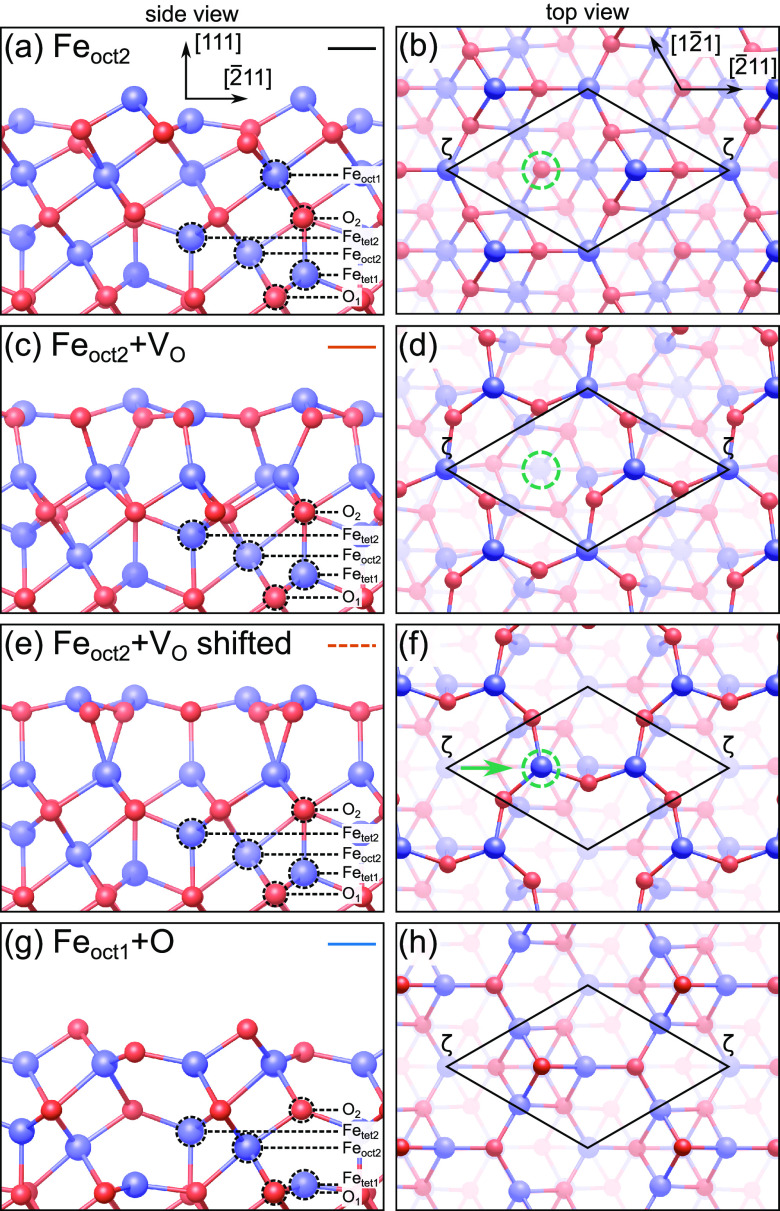
Reduced terminations of the Fe_3_O_4_(111) surface. Iron is blue (large), and oxygen is red (small). (a, b) The “standard” Fe_oct2_ termination. (c, d) The Fe_oct2_ termination with one additional oxygen vacancy at the site marked by a dashed green circle in (b, d). (e, f) Registry-shifted version of the Fe_oct2_+V_O_ structure, obtained by moving one surface iron as indicated by the green arrow in (f). (g, h) Relaxed Fe_oct1_ termination with iron trimers capped by an additional oxygen atom per unit cell. A (1 × 1) unit cell is indicated in black, with the corners at Fe_tet1_ positions (labeled as site ζ, see below). Line styles corresponding to Figure 2 are shown in the top-right corners of (a, c, e, g).

The first structure (corresponding to the solid orange line in Figure 2) is essentially an Fe_oct2_ termination with one additional surface oxygen vacancy per unit cell, as shown in Figure 3(c, d). The vacancy position is indicated by a dashed green circle in Figure 3(b, d). This modification yields lower surface energies than Fe_oct2_ under all conditions where a reduced termination is favorable over the Fe_tet1_ surface. Upon creation of the vacancy, the remaining surface oxygen atoms relax outward and each breaks one bond to an underlying Fe_oct1_ atom. This leaves the subsurface iron layer tetrahedrally coordinated. The reduced coordination of surface oxygen allows rotation of the surface FeO_3_ moieties, reducing the plane symmetry group from *p*3*m*1 to *p*3. As a result, the top Fe_oct2_ atoms gain the necessary space to relax further into the surface, forming an almost planar Fe_2_O_3_ layer. We denote this structure as the “Fe_oct2_+V_O_” termination.

The surface oxygen vacancy and the reduced coordination of the surface Fe_2_O_3_ layer facilitate a further modification, shown in Figure 3(e, f). The Fe_tet1_ atom [positioned at the unit cell corners in Figure 3(d)] can be moved laterally into the oxygen vacancy site, as indicated by the green arrow in Figure 3(f). The shift enables further relaxation of the surface and allows the subsurface iron tetrahedra to become less distorted. Nevertheless, this configuration (dashed orange line in Figure 2) is energetically less favorable than the Fe_oct2_+V_O_ without the registry shift. However, the energy difference depends somewhat on the theoretical setup (Figure S1): Using experimental Fe_3_O_4_ lattice constants (*a* = 5.94 Å), the registry shift would cost ∼5 meV/Å^2^, but this value is reduced to only ∼2 meV/Å^2^ for a slab constructed from a PBE+*U*-optimized bulk (*a* = 5.98 Å). The difference is likely due to an increased sensitivity of the surface Fe_2_O_3_ layer to strain: In all other structures considered here, suboptimal Fe–O distances due to in-plane strain can be compensated by expanding the structure in the out-of-plane direction with only minor changes to each atom’s environment. In contrast, the Fe_oct2_+V_O_ structure seems to favor coplanar iron and oxygen in the topmost layer, and reducing the lattice constant forces at least one iron atom farther out of the surface. In summary, the DFT results suggest that the registry shift is unfavorable, but the energetic differences are too small to unambiguously rule out either model. As will be discussed below, however, the Fe_oct2_+V_O_ model is in conflict with experimental data without the registry shift.

Finally, we report another competitive reduced reconstruction based on adding one oxygen atom per unit cell to the Fe_oct1_ termination. The structure after relaxation is shown in Figure 3(g, h), and a more comprehensive illustration of the relaxation and the spatial relationship to the Fe_tet1_ termination is given in Figure S2. Importantly, in addition to the capping oxygen atom, a subsurface oxygen atom breaks a bond to a subsurface Fe_tet1_ atom and relaxes to a 3-fold coordinated bridging site, such that the surface is terminated by two oxygen atoms per unit cell. This leaves one subsurface iron atom under-coordinated (three O neighbors), but this is compensated by the resulting near-perfect tetrahedral coordination of the three surface iron atoms. Despite being oxygen-terminated, this termination is still reduced with respect to bulk Fe_3_O_4_. All iron atoms in the surface Fe layer (formally Fe_oct1_, now tetrahedrally coordinated) exhibit a Bader charge of +1.28 *e*. In bulk-like layers, we find a charge disproportionation of ∼0.3 *e* resulting in Fe^2+^-like and Fe^3+^-like octahedral iron with Bader charges of 1.37–1.39 *e* and 1.67–1.70 *e*, respectively, in good agreement with previous results for bulk magnetite.^20^ For Fe_tet_ ions in bulk-like layers, which should always be in a 3+ state, we find a Bader charge of 1.62 *e*. Therefore, we assign the surface Fe_oct1_ cations to be in a 2+-like charge state. Interestingly, a similar Fe_oct1_+O model (without relaxation) was previously proposed by Lennie et al. for what is now considered the Fe_tet1_ termination^13^ but was subsequently discarded.

STM and ncAFM experiments were performed to complement the computational results. To ensure that our sample preparation yields surfaces comparable to the most recent literature, we first prepared a homogeneous (1 × 1)-periodic surface, corresponding to the previously reported Fe_tet1_ termination.^7,8,11^ Samples were sputtered (1 keV Ar^+^ ions, 10 min), annealed for 15 min in 10^–6^ mbar of O_2_ at 870–930 K, and then kept at the annealing temperature for another 5 min after evacuating O_2_ to ensure low residual oxygen pressure during cooling. This avoids the formation of oxygen-related defects.^7^ The best annealing temperature for producing defect-poor surfaces varied from sample to sample, most likely because our thermocouples were not mounted directly on the samples, causing some systematic error.

We then performed STM and ncAFM (Figure 4), as well as water temperature-programmed desorption (TPD) measurements (Figure S3) to confirm that our preparation of single crystal surfaces yields the same Fe_tet1_ termination as the thin film growth reported in ref (21). Both the STM images and the water TPD correspond well with previously published data.^11,13,21^ Bright features in the STM images are attributed to Fe_tet1_ atoms. The missing features have been attributed to adsorbates in cases where no feature is missing in empty-states STM and to Fe vacancies in cases where features are missing in both filled and empty-states STM.^11^ However, low-temperature STM and corresponding ncAFM images [Figure 4(c, d)] show that apparent vacancies which are seemingly identical in STM can also differ: The defect marked with the magenta arrow appears to show weak interaction in ncAFM, which may correspond to an iron vacancy. The vacancy-like feature in STM marked by the orange arrow shows a different interaction in ncAFM, possibly due to an adsorbate.

**Figure 4:**
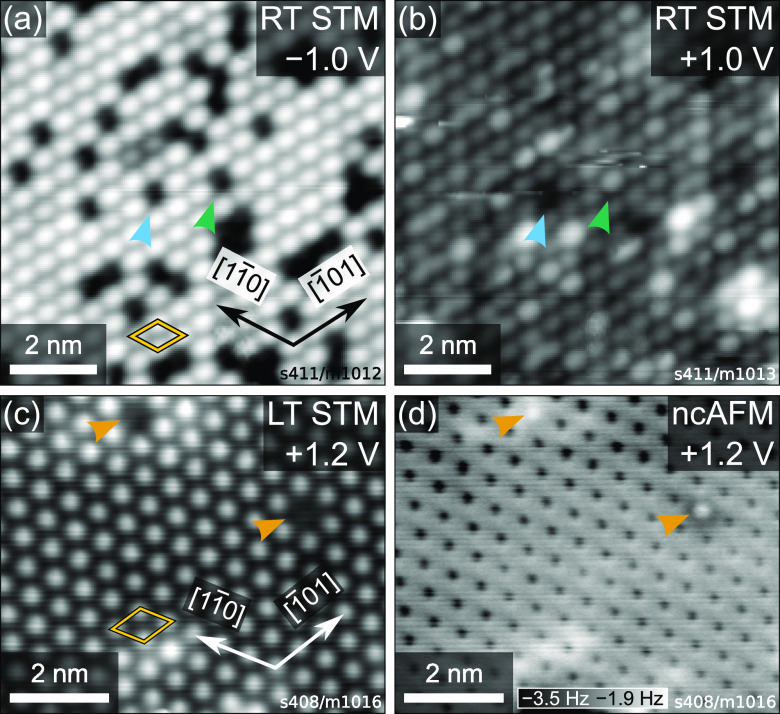
STM and ncAFM images of the Fe_3_O_4_(111) Fe_tet1_ termination. (a, b) Consecutive room-temperature STM images (*I*_tunnel_ = 0.1 nA) showing the same sample area, imaging (a) filled states (*U*_sample_ = −1.0 V) and (b) empty states (*U*_sample_ = +1.0 V). (c, d) Constant-height STM and ncAFM images acquired simultaneously at LN_2_ temperature with *U*_sample_ = +1.2 V.

Next, we address the termination frequently found to coexist with Fe_tet1_ areas on slightly reduced samples, which has previously been assigned as an Fe_oct2_ termination.^11,13^ Since our DFT results indicate that this assignment is incorrect, we will here refer to it simply as the “honeycomb termination” when describing experimental evidence, based on its appearance in scanning probe images. Note that this is different from the “biphase” reconstruction, which also has a honeycomb appearance, though at a much larger scale (∼5 nm periodicity).^14−17^

To obtain slightly reduced surfaces, samples were repeatedly sputtered (1 keV Ar^+^ ions, 10 min) and annealed in UHV (20 min at 870–930 K), with only the final annealing being performed in 10^–6^ mbar of O_2_. After oxygen annealing, the samples were kept at the annealing temperature for another 5 min to ensure low residual oxygen pressure during cooling. This generally resulted in surfaces exposing the Fe_tet1_ termination as well as patches of another termination with a honeycomb appearance in STM, as shown in Figure 5(a) and (d). When samples were overly reduced, they also exhibited patches of reduced “biphase” termination,^14−17^ which will not be directly addressed here. The few larger bright features visible in panel (a) are Pt clusters previously used for ncAFM tip preparation,^22^ which were subsequently encapsulated during annealing^23^ and remained in the subsurface even after more than 10 cycles of sputtering/annealing. The presence of these subsurface clusters does not affect the surface reconstruction outside the clusters’ immediate vicinity, as clearly seen in Figure 5(b). The STM appearance of both the honeycomb and Fe_tet1_ terminations in this data set is fully consistent with Pt-free data and with other images in the literature.

**Figure 5:**
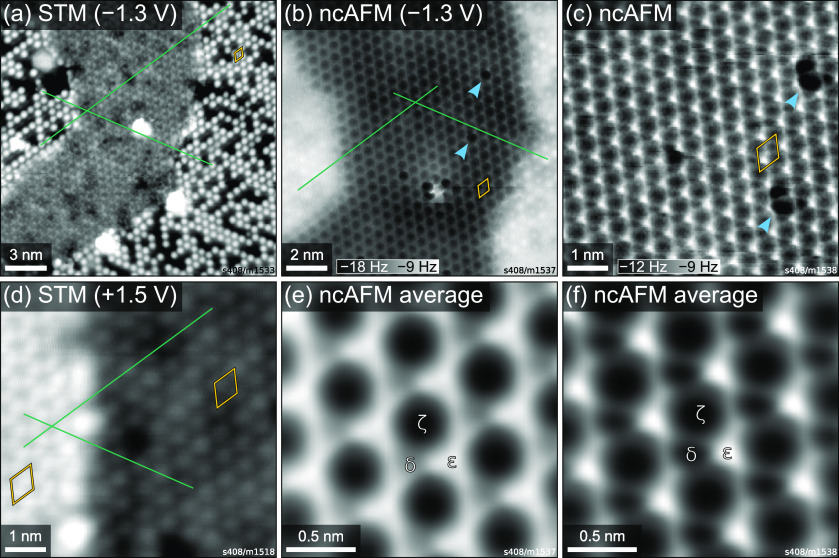
Low-temperature (*T* = 78 K) STM and ncAFM images of the “honeycomb” termination (assigned as Fe_oct2_ in previous works) formed under reducing conditions, coexisting with the Fe_tet1_ termination. (a, d) Constant-current STM images acquired at different positions on the sample with *I*_tunnel_ = 50 pA and bias voltages of (a) *U*_sample_ = −1.3 V and (d) *U*_sample_ = +1.5 V. (b, c) Constant-height ncAFM images of the same area as shown in (a) with higher magnification. A sample bias of −1.3 V was applied in (b); the image in (c) was taken without bias at a height 60 pm closer to the surface, with 400 pm oscillation amplitude in both cases. (e, f) Image averages over the unit cells in the honeycomb areas of panels (b) and (c), respectively. Green lines in panels (a), (b), and (d) are aligned with bright features in the Fe_tet1_ areas to highlight the relative positions of the features in the honeycomb areas. Blue arrows mark the same two defects in (b) and (c). (1 × 1) unit cells are marked in orange. Unit cell corners are placed at ζ sites, in registry with Fe_tet1_ atoms, both here and in Figure 3.

STM images of the honeycomb termination agree well with previously published results.^11,13^ While the Fe_tet1_ termination is characterized by one bright feature per unit cell, the honeycomb appearance results from two bright features in every unit cell. Point defects consisting of one missing feature are also observed in STM images of the honeycomb phase (Figure S4), as reported previously.^13^ Green lines in Figure 5(a) and (d) highlight the relative positions of bright features in the two terminations. In both cases, the Fe_tet1_ features are aligned with holes in the honeycomb phase. Furthermore, both STM images in Figure 5 show the two terminations at very similar apparent heights, with the Fe_tet1_ phase 25 pm above the honeycomb phase in Figure 5(a) (filled states, *U*_sample_ = −1.3 V) and 70 pm below the honeycomb phase in Figure 5(d) (empty states, *U*_sample_ = +1.5 V; line profile shown in Figure S4). Both of these values are much smaller than the height expected for a step between terraces of the same termination (485 pm). This bias dependence makes it seem likely that in both cases the geometric height of the two phases is similar and that the apparent height difference is caused mainly by differences in the electronic structure. Note however that this does not preclude small differences of the geometric height (e.g., additional atoms or a small interlayer distance). These data are in good agreement with the results by Lennie et al., who found the apparent height of the honeycomb phase to be 50 pm above that of the Fe_tet1_ phase at +2 V sample bias, and also report alignment of Fe_tet1_ features with holes in the honeycomb.^13^ Note that this is in conflict with the assignment of the honeycomb pattern as an Fe_oct2_ termination, which would require the Fe_tet1_ features to be aligned with one of the bright features of the honeycomb.

The assignment of a similar height for the two phases is corroborated by the constant-height ncAFM images shown in Figure 5(b). Both Figure 5(b) and (c) were taken on the same sample area as in Figure 5(a). In Figure 5(b), both the honeycomb and Fe_tet1_ phases are clearly resolved, indicating a similar height. Again, green lines indicate the relative positions of features in the two phases, and Fe_tet1_ atoms are in phase with a darker area in the honeycomb. For easier inspection, parts (e) and (f) of Figure 5 show averages over the unit cells of the honeycomb areas in parts (b) and (c), respectively. This suppresses noise and provides a clear resolution of three different 3-fold sites of the unit cell in both images, labeled as δ, ε, and ζ (following the nomenclature in ref (13)), where ζ is in phase with the Fe_tet1_ features. The appearance of ζ and ε as dark and bright is the same in both images, while δ appears with intermediate brightness in Figure 5(b) but is darker in Figure 5(c), where the tip is closer to the surface.

Overall, both the DFT and microscopy results show that the previous assignment of the honeycomb pattern as an Fe_oct2_ termination is incorrect. We find significantly lower surface energies for alternative terminations (Figure 2), and the alignment of the two phases in STM and ncAFM images (Figure 5) is in conflict with their interpretation as Fe_tet1_ and Fe_oct2_. This registry mismatch is already apparent in STM images published in previous studies.^11,13^ Some uncertainty had previously remained because true atomic heights cannot be accurately determined from the apparent heights in STM, but this ambiguity is removed by ncAFM, which rules out a large step between the Fe_tet1_ and honeycomb terminations in Figure 5. We therefore conclude that any viable model for the reduced termination must have the Fe_tet1_ sites aligned with holes of the honeycomb pattern.

We have introduced two models that fit this structural criterion, presented in Figure 3(e–h). First, the Fe_oct2_ termination can be modified by introducing one surface oxygen vacancy [Figure 3(c, d)] and then shifting the registry of the surface layer [Figure 3(e, f)]. We find this shift to be energetically unfavorable, but the energy difference found by DFT (2–5 meV/Å^2^, Figure S1) is too small to conclusively rule out the possibility. Since the registry shift moves the Fe_tet1_ atom away from its original position and leaves that site empty, the shifted structure is consistent with the observed alignment of Fe_tet1_ features with holes in the honeycomb. In contrast, without the registry shift, the Fe_oct2_+V_O_ termination leaves the lateral positions of surface iron atoms with respect to the Fe_tet1_ termination unchanged [Figure 3(c, d)] and is therefore still in conflict with the scanning-probe images.

The second viable model is a relaxed Fe_oct1_+O termination, which is favorable at higher oxygen chemical potential [Figure 3 (g, h)]. Here, the surface contains three symmetry-equivalent iron atoms per unit cell, which are brought into a near-perfect tetrahedral coordination with two capping oxygen atoms. Unlike the other models, bright features in the honeycomb pattern seen in STM would here be associated with surface oxygen, rather than iron. Simulated STM images of the Fe_oct1_+O termination (Figure S5) confirm this assignment. The point defects observed in the STM images (Figure S4) would then most likely correspond to oxygen vacancies. After relaxation, the topmost oxygen atom in the Fe_oct1_+O model is at almost the same height as the surface iron atom in the Fe_tet1_ termination (Δ*z* = 0.17 Å; see Table S1), in good agreement with the appearance in STM and ncAFM.

While the scanning-probe images in Figure 5 could be rationalized by either the Fe_oct1_+O or the registry-shifted Fe_oct2_+V_O_ termination, the oxygen-capped Fe_oct1_+O model is more plausible based on other experimental evidence. First, its predicted stability region is adjacent to that of the Fe_tet1_ surface. If the honeycomb termination would correspond to the Fe_oct2_+V_O_ termination, then it should be possible to also observe separate regions of the Fe_oct1_+O termination, i.e., two different honeycomb patterns. This does not appear to be the case, which suggests that the honeycomb phase corresponds to the model that is stable at a higher oxygen chemical potential. Second, there is previous experimental evidence that the honeycomb regions are much less reactive to adsorbates than the Fe_tet1_ surface.^24,25^ This would agree well with the oxygen-terminated Fe_oct1_+O model, in which all surface iron is fully 4-fold coordinated. In contrast, higher reactivity than the Fe_tet1_ surface would be expected for any Fe_oct2_ or Fe_oct2_+V_O_ termination, since these expose two under-coordinated iron atoms per unit cell. CO stretching frequencies for adsorption on the Fe_oct2_ ion have been calculated^8^ but were never observed in infrared reflection absorption spectroscopy (IRAS) experiments,^5,8^ suggesting that such sites do not exist or do not accommodate CO. Finally, three distinct features δ, ε, and ζ are observed in ncAFM images (Figure 5). This contrast can be interpreted as interaction with two surface atoms at different heights positioned at δ and ε and no interaction at the ζ site. This fits the two capping oxygen atoms in the Fe_oct1_+O model, which are clearly at different heights. On the other hand, the surface iron atoms of the Fe_oct2_+V_O_ surfaces are at very similar heights, and one would expect similar contrast in ncAFM. Therefore, we conclude that the honeycomb regions are best explained by the Fe_oct1_+O model.

A further attractive aspect of the improved models for reduced surface terminations is that this shifts the transition point between the Fe_tet1_ surface and the best reduced model to a higher oxygen chemical potential. In the surface phase diagram shown in Figure 2, the transition is predicted at Δμ_O_ = −2.6 eV. This is still quite low but achievable by UHV annealing, unlike the −3.0 eV required for a transition to Fe_oct2_. The model therefore helps to understand why patches of the honeycomb phase are commonly observed when flashing or postannealing samples after oxygen has been pumped out, which puts the sample at a low but somewhat ill-defined chemical potential.

It is important to note that a monophase termination of Fe_3_O_4_(111) with the honeycomb structure cannot be prepared. When samples are reduced further, they instead restructure into the so-called “biphase” termination. However, the new motifs identified here may also be helpful in explaining the constituent structures of the biphase. In our Fe_oct2_+V_O_ models [Figure 3(c-f)], the kagome Fe_oct1_ layer is transformed to a tetrahedral coordination, with only one bond per iron atom to the Fe_2_O_3_ layer. This allows for significant flexibility in the placement of the adlayer, as evidenced by the low energy cost of the registry shift. We tentatively propose that this same tetrahedrally coordinated kagome layer could support either a range of different reduced (1 × 1) structures^14−17^ or an FeO-like adlayer in a moiré structure.^18^ Following the 18:17 relationship between the substrate and the adlayer proposed by Spiridis et al.,^18^ a (17 × 17) supercell of a wüstite-based FeO or Fe_2_O_2_ layer could be attached to a (9 × 9) supercell of the Fe_3_O_4_(111) surface with only ∼3% lattice strain of the adlayer. The apparently flexible bond angles of the tetrahedrally coordinated iron in the kagome Fe_oct1_ layer could conceivably accommodate such an attachment. Of course, significant modifications of such structures and variations in stoichiometry may be necessary to explain the range of different morphologies observed for biphase structures.^14−18^ If the “biphase” termination does in fact contain a kagome layer with reduced coordination to oxygen, it may be interesting to investigate whether the weaker linking between iron atoms gives rise to clean kagome bands.^26^

In conclusion, a combination of DFT calculations and scanning-probe methods has allowed us to shed new light on the structural motifs observed on the Fe_3_O_4_(111) surface under reducing conditions. Both the experimental evidence and DFT results conclusively rule out the previously accepted Fe_oct2_ model. Somewhat counterintuitively, we conclude that an oxygen-terminated reconstruction is formed under reducing conditions, which helps explain the relatively inert behavior observed in experiment.

## Experimental and Computational Methods

Experiments were performed on natural single crystals (SurfaceNet GmbH, <0.3° miscut). Samples were cleaned by cycles of 1 keV Ar^+^ or Ne^+^ sputtering and annealing in oxygen and UHV as described in the main text until free from contaminants as judged by X-ray photoelectron spectroscopy (XPS). Three UHV setups were used in this study: Room-temperature STM was performed in a UHV setup equipped with a non-monochromatic Al Kα X-ray source (VG), a SPECS Phoibos 100 analyzer for XPS, and a μ-STM. Low-temperature STM and ncAFM were performed in a second setup using an Omicron LTSTM equipped with a Qplus sensor and an in-vacuum preamplifier.^27^ Finally, to confirm that the single crystal surfaces are equivalent to thin films studied in previous work, high-quality TPD and XPS data were acquired in a molecular beam setup designed to study the reactivity of oxide single crystals, described in detail in ref (28). Samples studied in the ncAFM chamber, which is not equipped with XPS, were first examined in the room-temperature STM chamber and then cleaned again after transfer. Scanning probe images were corrected for distortion and creep of the piezo scanner, as described in ref (29). Image averages in Figure 5 were obtained by algorithmically detecting each ζ site of the honeycomb pattern and then averaging over 2 × 2 nm^2^ image areas centered at those sites.

The Vienna ab initio Simulation Package (VASP)^30,31^ was used for all calculations, with near-core regions described by the projector augmented wave method.^32,33^ A Γ-centered *k*-mesh of 7 × 7 × 1 was used for all (1 × 1) slabs, and the plane wave basis set cutoff energy was set to 550 eV. Calculations were performed at the PBE+*U* level,^34,35^ with an on-site Coulomb repulsion term *U*_eff_ = 3.61 eV based on previous work.^36^ Slabs were relaxed until the residual forces acting on the ions were smaller than 0.02 eV/Å. Surface phase diagrams were derived following the approach described by Reuter and Scheffler,^37^ using bulk Fe_3_O_4_ and a free oxygen molecule in the triplet state as references. Simulated STM images were created by using the Tersoff–Hamann approximation in constant-height mode.^38^ The charge states of iron cations were evaluated by using the Bader approach.^39−41^ Reported Bader charge values are the differences between the 8 valence electrons considered in the calculations and the total projected charge in the Bader volume.

Slabs were constructed from an experimentally determined bulk unit cell (*Fd*3̅*m*, *a* = 8.396 Å, JCPDS file^42^ 19-629). We primarily used asymmetric slabs containing 15–17 Fe layers depending on the surface termination, with a vacuum gap of at least 15 Å and applying dipole corrections as implemented in VASP. An Fe_oct2_ termination was used at the bottom of the slab such that the Fe_tet1_-terminated slab is stoichiometric overall. The bottom 7 Fe layers and corresponding oxygen were kept fixed. Slabs yielding the lowest surface energies were also recalculated based on a PBE+*U*-optimized bulk, which overestimates the lattice constant by ∼0.7%. Similarly, we also tested the most relevant terminations on symmetric slabs (13–17 Fe layers). Relative surface energies changed slightly in both cases but did not significantly affect the conclusions. A comparison of the surface phase diagrams based on the three different setups is shown in Figure S1.

## Supporting Information Available

Additional figures showing surface phase diagrams for different theoretical slab setups, illustration of the relationship between Fe_tet1_ and Fe_oct1_+V_O_ terminations, water TPD spectra, STM image showing point defects in the honeycomb termination, and STM simulations and table listing relative geometric heights of surface atoms in various terminations (PDF)
DFT-optimized structure file for O_1_ (CIF)
DFT-optimized structure file for O_1_-relaxed (CIF)
DFT-optimized structure file for Fe_oct1_+O (CIF)
DFT-optimized structure file for Fe_oct2_ (CIF)
DFT-optimized structure file for Fe_oct2_+V_O_ (CIF)
DFT-optimized structure file for Fe_oct2_+V_O_ shifted (CIF)
DFT-optimized structure file for Fe_tet1_ (CIF)
DFT-optimized structure file for Fe_oct1_+V_Fe_ (CIF)

## Supplementary Material

jz3c00281_si_001.pdf

jz3c00281_si_002.cif

jz3c00281_si_003.cif

jz3c00281_si_004.cif

jz3c00281_si_005.cif

jz3c00281_si_006.cif

jz3c00281_si_007.cif

jz3c00281_si_008.cif

jz3c00281_si_009.cif
