# Supplementary material for: Oxygen-Terminated (1 × 1) Reconstruction of Reduced Magnetite Fe3O4(111)
Source: J Phys Chem Lett. 2023 Mar 28;14(13):3258–65. doi: 10.1021/acs.jpclett.3c00281 (PMC10084462; doi:10.1021/acs.jpclett.3c00281)
Supplement: Supplementary file 1 — jz3c00281_si_001.pdf [file jz3c00281_si_001.pdf]

Supporting Information

Oxygen-Terminated (1 × 1) Reconstruction of Reduced Magnetite $Fe_3O_4(111)$

*Florian Kraushofer[1,†], Matthias Meier[1,2], Zdeněk Jakub[1,‡], Johanna Hütner[1], Jan Balajka[1], Jan Hulva[1], Michael Schmid[1], Cesare Franchini[2,3], Ulrike Diebold[1], Gareth S. Parkinson[1]**

[1] Institute of Applied Physics, Technische Universität Wien, Wiedner Hauptstraße 8-10/E134, 1040 Wien, Austria

[2] University of Vienna, Faculty of Physics and Center for Computational Materials Science, 1090 Wien, Austria

[3] Alma Mater Studiorum, Università di Bologna, 40127 Bologna, Italy

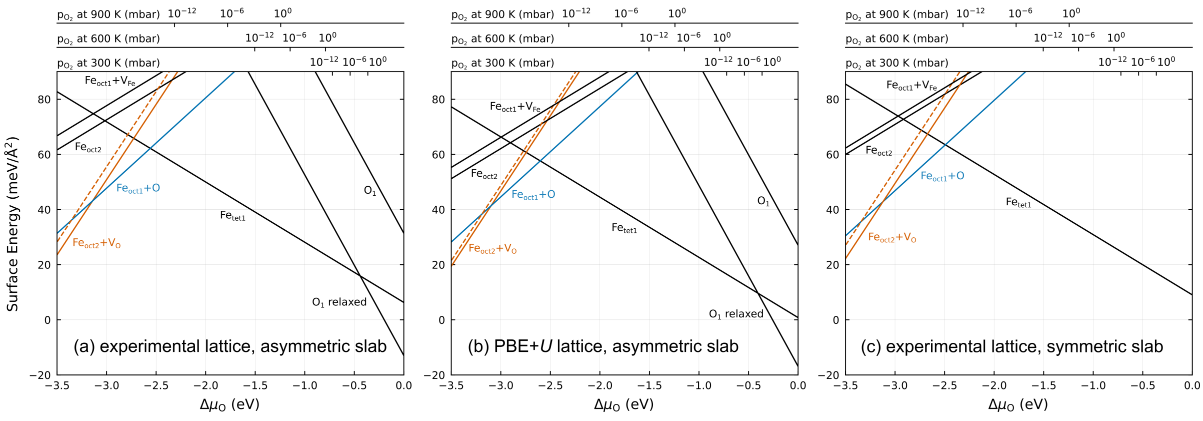

Figure S1: Surface energies of different terminations as a function of the oxygen chemical potential, obtained for different slabs. (a) Asymmetric slab using the experimental lattice constant, also shown as Figure 2 in the main manuscript. (b) Asymmetric slab based on a PBE+*U*-optimized bulk cell. (c) Symmetric slab using the experimental lattice constant. The top axes indicate the pressures corresponding to the chemical potential at the given temperatures. Colored lines are new models introduced in this work, black lines correspond to terminations considered in previous work. "O_1 relaxed" is the modified O_1 termination introduced in ref. 1, while all other models can be found in ref. 2.

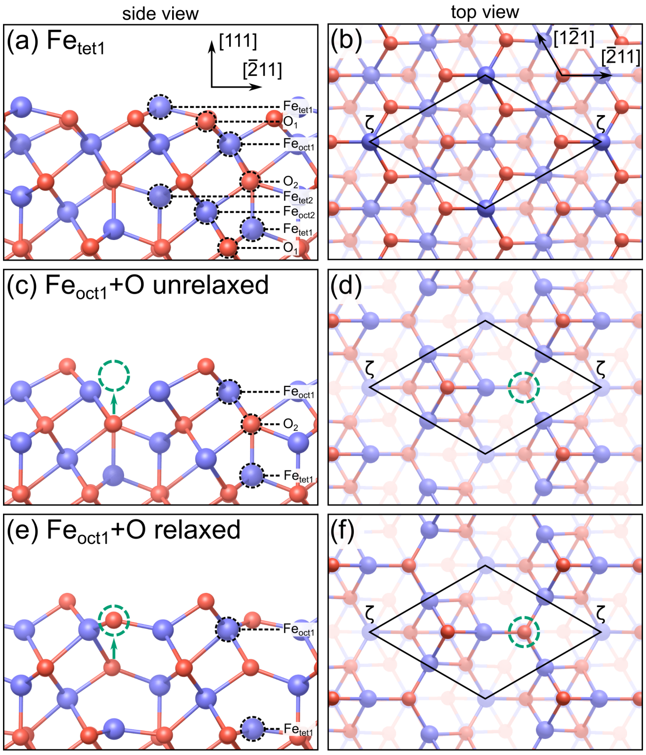

Figure S2: Illustration of the relationship between the Fe_{tet1} and the Fe_{oct1}+O terminations. Iron is blue (large), oxygen is red (small). (a, b) The “standard” Fe_{tet1} termination. (c, d) The Fe_{oct1} termination with iron trimers capped by an additional oxygen atom per unit cell, as cut from the bulk structure. (e, f) The same Fe_{oct1}+O termination after DFT relaxation, as shown in Figure 3. One subsurface oxygen atom per unit cell breaks its bond to an underlying Fe_{tet1} and relaxes out of the surface, as indicated by the green arrows and dashed green circles in panels (c-f). The unit cell position relative to the bulk is the same in panels (b), (d), and (f), with the corners at Fe_{tet1} sites (ζ sites).

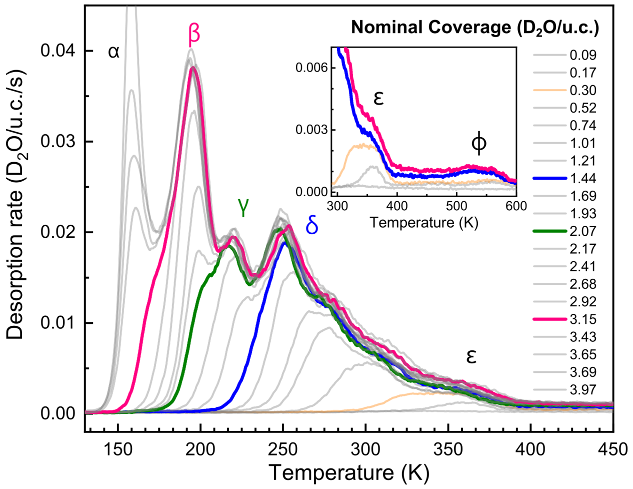

Figure S3: Experimental TPD spectra (1 K/s heating rate) obtained for initial D_2O coverages ranging from 0 to 4 molecules per Fe_{tet1}-terminated Fe_3O_4(111)-(1 × 1) unit cell. Water TPD was acquired to confirm that our preparation of single crystal surfaces yields the same Fe_{tet1} termination as the thin film growth reported in ref. 3. A magnified view of desorption peaks ε and Φ at higher temperatures is shown in the inset. The colored curves indicate the coverages at which a particular desorption feature saturates. The nominal coverage given in the figure legend may underestimate the actual coverage by up to 10%.

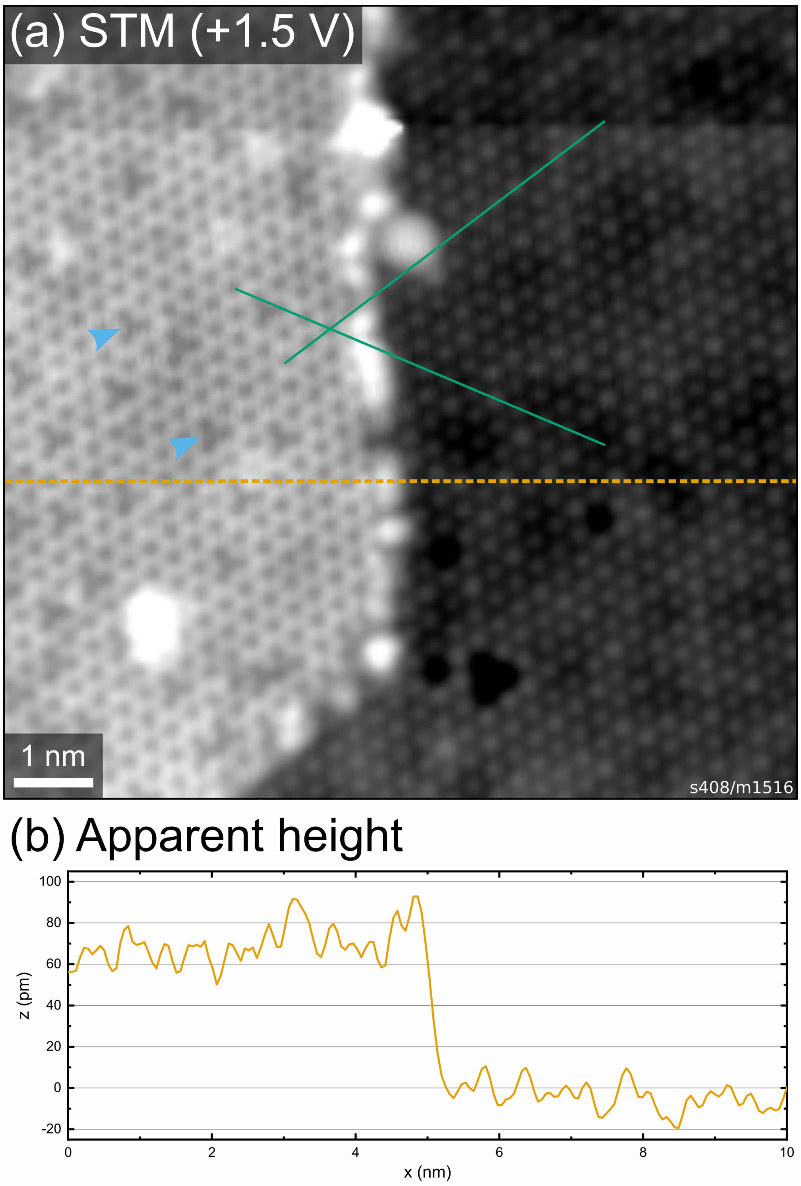

Figure S4: (a) Low-temperature (T = 78 K) STM image (I_{tunnel} = 50 pA, U_{sample} = +1.5 V) of the “honeycomb” termination formed under reducing conditions, coexisting with the Fe_{tet1} termination. Green lines are aligned with bright features in the Fe_{tet1} areas to highlight relative positions of features in the honeycomb areas. Blue arrows mark point defects in the honeycomb phase, consisting of one missing bright feature in the ε site.
(b) Apparent-height profile along the dashed orange line in (a).

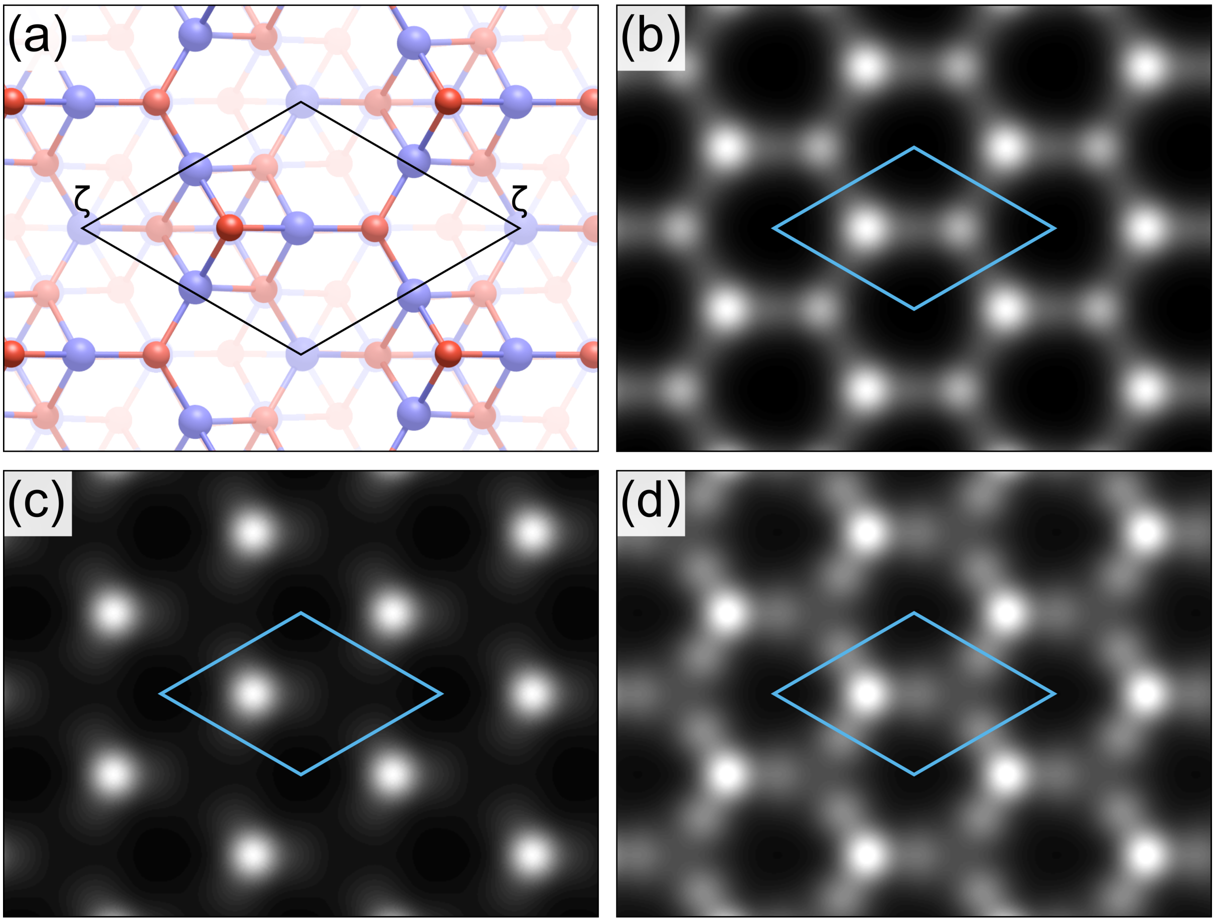

Figure S5: (a) Relaxed Fe_{oct1}+O termination as shown in Figure 3 (h) and Figure S2 (f), with the unit cell drawn in black and unit cell corners at Fe_{tet1} sites (site ζ in Figure 5). (b-d) Simulated constant-height STM images of the Fe_{oct1}+O termination with sample bias voltages of (b) −2 V, (c) +2 V and (d) +0.5 V. The position and orientation of the blue unit cells is the same as for the black unit cell in (a). The typical honeycomb contrast is reproduced in (b) and (d), while (c) shows only one feature per unit cell (again out of registry with the Fe_{tet1} sites), as is also sometimes observed in experiment.[4, 5] Bright features correspond to oxygen atoms in (b) and (c), while both iron and oxygen atoms contribute to the rings in (d).

**Δz [Å]**	Fe_{tet1}	Fe_{oct2}+V_O	Fe_{oct2}+V_O shifted	Fe_{oct1}+O
surface Fe	0.00	+0.78 / +0.40	+0.83 / +0.78	−1.24
surface O	−0.43	+0.27	+0.41	−0.17 / −0.87

Table S1: Relative height (coordinate perpendicular to the surface) in Å with respect to surface iron atom in the Fe_{tet1} termination for surface iron and oxygen atoms in selected terminations.

References

(1) Creutzburg, M.; Sellschopp, K.; Tober, S.; Grånäs, E.; Vonk, V.; Mayr-Schmölzer, W.; Müller, S.; Noei, H.; Vonbun-Feldbauer, G. B.; Stierle, A. Heterogeneous Adsorption and Local Ordering of Formate on a Magnetite Surface. *J. Phys. Chem. Lett.* **2021**, *12*, 3847-3852. DOI: 10.1021/acs.jpclett.1c00209.

(2) Noh, J.; Osman, O. I.; Aziz, S. G.; Winget, P.; Brédas, J.-L. Magnetite Fe_3O_4 (111) Surfaces: Impact of Defects on Structure, Stability, and Electronic Properties. *Chem. Mater.* **2015**, *27*, 5856-5867. DOI: 10.1021/acs.chemmater.5b02885.
(3) Mirabella, F.; Zaki, E.; Ivars-Barceló, F.; Li, X.; Paier, J.; Sauer, J.; Shaikhutdinov, S.; Freund, H. J. Cooperative Formation of Long-Range Ordering in Water Ad-layers on Fe_3O_4(111) Surfaces. *Angew. Chem. Int. Ed.* **2018**, *57*, 1409-1413. DOI: 10.1002/anie.201711890.
(4) Lennie, A.; Condon, N.; Leibsle, F.; Murray, P.; Thornton, G.; Vaughan, D. Structures of Fe_3O_4(111) surfaces observed by scanning tunneling microscopy. *Phys. Rev. B* **1996**, *53*, 10244. DOI: 10.1103/physrevb.53.10244.
(5) Shimizu, T. K.; Jung, J.; Kato, H. S.; Kim, Y.; Kawai, M. Termination and Verwey transition of the (111) surface of magnetite studied by scanning tunneling microscopy and first-principles calculations. *Phys. Rev. B* **2010**, *81*, 235429. DOI: 10.1103/PhysRevB.81.235429.
